# Comparative analysis of chloroplast genomes of seven *Juniperus* species from Kazakhstan

**DOI:** 10.1371/journal.pone.0295550

**Published:** 2024-01-25

**Authors:** Shyryn Almerekova, Moldir Yermagambetova, Smatulla Jumanov, Saule Abugalieva, Yerlan Turuspekov

**Affiliations:** 1 Molecular Genetics Laboratory, Institute of Plant Biology and Biotechnology, Almaty, Kazakhstan; 2 Faculty of Biology and Biotechnology, Al-Farabi Kazakh National University, Almaty, Kazakhstan; 3 Aksu-Zhabagly Nature Reserve, Zhabagly, Turkestan Region, Kazakhstan; Institute for Biological Research, University of Belgrade, SERBIA

## Abstract

*Juniperus* species are shrubs or trees in the family Cupressaceae that play an important role in forest ecosystems. In this study, we report the complete sequences of the plastid (pt) genomes of five *Juniperus* species collected in Kazakhstan (*J*. *communis*, *J*. *sibirica*, *J*. *pseudosabina*, *J*. *semiglobosa*, and *J*. *davurica*). The sequences of the pt genomes of the five species were annotated in addition to two full pt genome sequences from *J*. *sabina* and *J*. *seravschanica*, which we have previously reported. The pt genome sequences of these seven species were compared to the pt genomes of *Juniperus* species available in the public NCBI database. The total length of the pt genomes of *Juniperus* species, including previously published pt genome data, ranged from 127,469 bp (*J*. *semiglobosa*) to 128,097 bp (*J*. *communis*). Each *Juniperus* plastome consisted of 119 genes, including 82 protein-coding genes, 33 transfer RNA and 4 ribosomal RNA genes. Among the identified genes, 16 contained one or two introns, and 2 tRNA genes were duplicated. A comparative assessment of pt genome sequences suggested the identification of 1145 simple sequence repeat markers. A phylogenetic tree of 26 *Juniperus* species based on the 82 protein-coding genes separated the *Juniperus* samples into two major clades, corresponding to the *Juniperus* and *Sabina* sections. The analysis of pt genome sequences indicated that *accD and ycf2* were the two most polymorphic genes. The phylogenetic evaluation of 26 *Juniperus* species using these two genes confirmed that they can be efficiently used as DNA barcodes for phylogenetic analyses in the genus. The sequenced plastomes of these *Juniperus* species have provided a large amount of genetic data that will be valuable for future genomic studies of this genus.

## Introduction

*Juniperus* L. is a genus in the family Cupressaceae that is mainly distributed throughout the Northern Hemisphere, from sea level to above the timberline. There are approximately 75 species in the genus, which belong to three different sections: *Sabina*, *Juniperus*, and *Caryocedrus* [1]. Species in the genus are widely distributed in dry foothills or at mid to high altitudes of Central Asian mountains, including Kazakhstan [2]. In Kazakhstan, the genus representatives are mainly found on dry mountain slopes and in alpine and subalpine belts [3, 4]. The country has eight species of *Juniperus*, including *J*. *communis* L., *J*. *sibirica* Burgsd., *J*. x *media* Dmitr., *J*. *pseudosabina* Fisch. et C.A. Mey., *J*. *sabina* L., *J*. *semiglobosa* Regel., *J*. *seravschanica* Kom. [5], and *J*. *davurica* Pall. [6]. Two of those species, *J*. *communis* and *J*. *sibirica*, belong to J. sect. *Juniperus*; the remaining six species belong to J. sect. *Sabina* [1]. *Juniperus* species play an important ecological role in the formation of mountain forest ecosystems as well as hydrobiological regulation and erosion prevention [1]. *Juniperus* species are highly significant in medicine and are used in perfumery and cosmetics [7–9]. Many chemical substances have been extracted from different *Juniperus* species, including cedrol [10, 11], glucosides [12, 13], polyphenols [14, 15], terpenoids, and tannins [1, 14, 16].

The genetic diversity of the species in the genus has been thoroughly studied using different types of DNA markers. In particular, markers such as random amplified polymorphic DNA (RAPD) [17–20], inter-simple sequence repeats (ISSR) [19, 21, 22], amplified fragment length polymorphisms (AFLP) [23–25], and single-nucleotide polymorphisms (SNP) [26] have been widely used to evaluate the genetic diversity of *Juniperus*. In the last 20 years, simple sequence repeats (SSRs), or microsatellites, have become the most popular marker; these are extensively employed in the population genetics of *Juniperus* species [27–31]. However, the taxonomic relationships of many *Juniperus* species are dubious due to their complex morphological characteristics [32, 33]. For example, the species *J*. *communis* includes several morphological forms, which has led to a complex taxonomy in this species [34]. Another example is *J*. *excelsa* M. Bieb, which consists of morphologically similar species such as *J*. *excelsa*, *J*. *polycarpos*, and *J*. *seravschanica* [35]. A taxonomic reassessment of *J*. *turkestanica* and *J*. *pseudosabina* from Central Asia was conducted by Adams and Turuspekov (1998) [18] using RAPD markers. The analysis, based on less reliable RAPD markers [36], revealed that *J*. *turkestanica* and *J*. *pseudosabina* belong to a single species named *J*. *pseudosabina*. Therefore, further clarification of the phylogeny requires new molecular tools for more accurate resolution in the genus. The application of next-generation sequencing (NGS) technologies may provide further resources for genetic diversity assessments of *Juniperus* species. The rapid advancements in NGS technologies have allowed us to quickly and inexpensively obtain genome sequences [37, 38]. One promising direction in the genomic study of *Juniperus* is the evaluation of the genetic variation in plastid genomes. The *Juniperus* plastid genome is a circular molecule with a size of around 130,000 bp. Due to the small sizes of plastid genomes and their conserved gene content, structure, and uniparentally inherited characteristics, they may be informative when assessing the genetic diversity and phylogenetic relationships within the genus [39]. For instance, plastid genome markers (*trnT-trnF*, *trnS-trnG*, *trnC-trnD*, *matK*, *rbcL*, *trnL*, *psbD*, *psbM*, and *petN*) have been successfully used to resolve phylogenetic relationships in the *Juniperus* genus [40–43].

Recently, promising steps have been accomplished in the characterization of the complete pt genomes of *Juniperus* species from China [44–50], Pakistan [51], and Kazakhstan [52, 53]. A complete pt genome comparison of four *Juniperus* species from China has been published [54]. Chen et al. (2022) [54] reported that five significantly divergent regions, including *accD*, *accD-rpl2*, *ycf1*, *ycf2*, and *rrn23-rrn4*.*5*, could be used as DNA barcodes to identify relationships between different species and potential genetic markers. The authors concluded that further samples were needed for a reliable extrapolation of the phylogenetic relationships of Cupressaceae [54]. Thus, assessing genetic variations in newly sequenced pt genomes of the genus may be highly informative for studies of taxonomic relationships with higher precision. In this work, we report a comparison of the complete pt genome sequences of seven *Juniperus* species from Kazakhstan and discuss the possibilities for generating molecular markers for a phylogenetic analysis of the genus.

## Materials and methods

### Plant material and DNA extraction

The leaves of five *Juniperus* species were collected from different geographical locations in Kazakhstan in 2017 and 2020–2021 (Table 1) and dried in silica gel for DNA isolation. The permission for collecting plant material of Red Book species *J*. *seravschanica* was obtained from the Forestry and Wildlife Committee Ministry of Ecology, Geology and Natural Resources of the Republic of Kazakhstan. Total DNA was extracted from dried leaves using the cetyltrimethylammonium bromide (CTAB) method [55]. The quality and quantity of the isolated DNA of the *Juniperus* samples were evaluated using agarose gel electrophoresis and a NanoDrop 2000 spectrophotometer (Thermo Fisher Scientific Inc., USA).

**Table 1:** Geographical locations of the collected *Juniperus* samples.

#	NCBI accession number	Species	Collected date	Collected place	Coordinates	Section
1	OQ644240	*J*. *communis*	10.09.2021	Akmola region, Burabai State National Natural Park, the vicinity of the lake Lebyazhye	53.020000, 70.174111 467 m a.s.l.	*Juniperus*
2	OQ644239	*J*. *sibirica*	27.09.2020	Almaty region, Ile-Alatau National Park, Kim Asar gorge	43.162500, 77.093889 2264 m a.s.l.	*Juniperus*
3	OQ644236	*J*. *davurica*	19.05.2017	East Kazakhstan region, Kokpektinsky district, foothills of the Kalbinsky ridge	49.280278, 83.121111 1230 m a.s.l.	*Sabina*
4	OQ644238	*J*. *pseudosabina*	12.05.2017	Almaty region, Ile-Alatau National Park, Big Almaty gorge	43.044500, 76.978500 2714 m a.s.l.	*Sabina*
5	OQ644237	*J*. *semiglobosa*	12.05.2021	Turkestan region, Sairam- Ugam state national natural park, Sairam gorge	42.167333, 70.416583 1845 m a.s.l.	*Sabina*

### Plastid genome sequencing, assembly, and annotation

Complete pt genome paired-end sequencing was performed using the Illumina NovaSeq 6000 platform (Illumina Inc., USA) at Macrogen Inc. (Seoul, Republic of Korea). DNA that passed quality control DNA was used for library construction using a TruSeq Nano DNA Kit (Illumina Inc., USA). The generated raw read FASTQ format files were used for the genome assembly. The raw data were checked using FastQC and trimmed using Trimmomatic tools [56]. Contigs were assembled from the trimmed reads using the SPAdes 3.13.0 [57] assembler approach. The assembled pt genomes were annotated using PGA [58] and GeSeq [59]. The graphical map of the plastid genomes of *Juniperus* species was generated using Organellar Genome DRAW (OGDRAW) v1.3.1 [60].

### Genome comparisons, SSRs, and repeat analyses

The nucleotide diversity (Pi) of the seven complete pt genomes of the *Juniperus* species was calculated using the DnaSP (DNA Sequence Polymorphism) package, with a 200 bp step size and a 600 bp window length [61]. Simple sequence repeats (SSRs) were detected using MISA software [62] with the following thresholds: eight for mononucleotide repeats, four for dinucleotide repeats, four for trinucleotide repeats, and three for tetranucleotide, pentanucleotide, and hexanucleotide repeats. The forward (F), reverse (R), and palindromic (P) repeat elements were identified using the PERuter web- based program [63] with the following parameter settings: Hamming distance = 3 and minimum repeat size = 30 bp. In addition, each of the *Juniperus* plastid genomes was searched for tandem repeats using the Tandem Repeats Finder program [64] with the default settings.

### Phylogenetic analysis

The concatenated sequences of 82 common protein-coding genes of 26 *Juniperus* samples were used for the phylogenetic analysis. *Hesperocyparis stephensonii* and *Cupressus torulosa* was used as the outgroup. The nucleotide sequences of the common protein-coding genes of five plastomes of *Juniperus* species from Kazakhstan were obtained in this study. The remaining 23 samples, including the outgroup, were extracted from the database of the National Center for Biotechnology Information (NCBI). Based on the Akaike information criterion, the best nucleotide substitution model (GTR+I+G) was calculated in jModelTest 2.1.10 [65]. Bayesian inference (BI) was carried out in MrBayes 3.2.7 [66]. FigTree 1.4.4 (http://tree.bio.ed.ac.uk/software/figtree/) was used to visualize and refine the BI-based phylogenetic tree. The maximum likelihood (ML) method [67] based tree was constructed using the MEGA 11 package [68] with 1000 bootstrap replicates. The newly obtained pt genome nucleotide sequences of the *Juniperus* species from Kazakhstan were deposited in GenBank. The accession numbers are listed in Table 1.

## Results

### Features of the plastomes of *Juniperus* species

DNA libraries of pt genomes from five *Juniperus* species (*J*. *communis*, *J*. *sibirica*, *J*. *davurica*, *J*. *pseudosabina*, and *J*. *semiglobosa*) were sequenced using the Illumina NovaSeq 6000 sequencing platform and compared with two previously reported pt genomes of *J*. *sabina* and *J*. *seravschanica* that had been collected in Kazakhstan. The largest amount of raw data in the compared pt genomes was in *J*. *pseudosabina* (4.88 GB), followed by *J*. *davurica* (4.38 GB), *J*. *sibirica* (4.37 GB), *J*. *semiglobosa* (4.18 GB), *J*. *sabina* (3.68 GB), *J*. *seravschanica* (3.27 GB), and *J*. *communis* (3.0 GB). The overall GC content of the assembled pt genomes of *J*. *communis*, *J*. *sibirica*, *J*. *pseudosabina*, *J*. *semiglobosa*, *J*. *davurica*, *J*. *sabina*, and *J*. *seravschanica* were 34.87%, 34.87%, 34.98%, 34.93%, 34.86%, 34.36%, and 34.45%, respectively (Table 2).

**Table 2:** Comparative characteristics of pt genomes of seven studied *Juniperus* species.

	NCBI accession number	Genome size (bp)	GC content, %	Coding genes	tRNA	rRNA	CDS total length (bp)	CDS length / Genome (%)
*J*. *communis*	OQ644240	128,097	34.87	82	33	4	81164	63.4
*J*. *sibirica*	OQ644239	128,046	34.87	82	33	4	81165	63.4
*J*. *davurica*	OQ644236	128,021	34.86	82	33	4	81144	63.4
*J*. *pseudosabina*	OQ644238	128,071	34.98	82	33	4	81248	63.4
*J*. *semiglobosa*	OQ644237	127,469	34.93	82	33	4	81142	63.7
*J*. *sabina*	OL467323	127,646	34.36	82	33	4	81110	63.5
*J*. *seravschanica*	OL684343	127,609	34.45	82	33	4	81139	63.6

**Fig 1:**
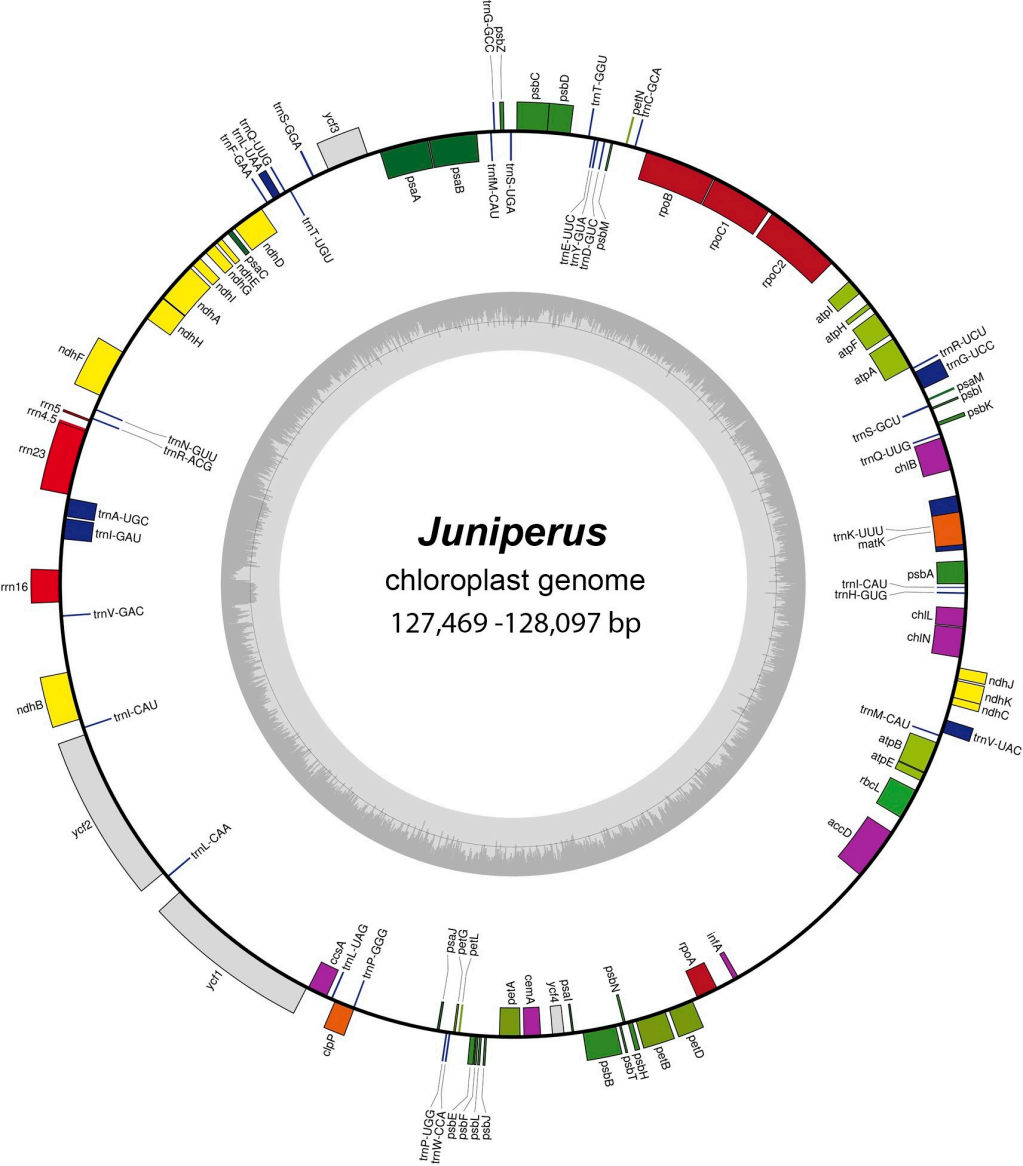
Gene map of the pt genomes of *Juniperus* species.

The assembled lengths of the pt genomes ranged from 127,469 bp in *J*. *semiglobosa* to 128,097 bp in *J*. *communis*. The consensus circular gene map of the complete pt genomes of *Juniperus* species from Kazakhstan is shown in Fig 1. Individual circular maps for the five newly generated pt genomes are provided in S1 Fig. The nucleotide sequences of the complete pt genome data were deposited in the NCBI database. The accession numbers are provided in Tables 1–3.

**Table 3:** List of genes in pt genomes of *J*. *communis*, *J*. *sibirica*, *J*. *pseudosabina*, *J*. *semiglobosa*, and *J*. *davurica*. One or two asterisks indicate one or two intron-containing genes, respectively; (x2) indicates the duplicated genes.

Category	Group of genes	Name of genes
Self-replication	Transfer RNA	trnA-UGC*, *trnC-GCA*, *trnD-GUC*, *trnE-UUC*, *trnF-GAA*, *trnfM-CAU*, *trnG-GCC*, trnG- UCC*, *trnH-GUG*, *trnI-CAU (x2)*, trnI-GAU*, trnK-UUU*, *trnL-CAA*, trnL-UAA*, *trnL-UAG*, *trnM-CAU*, *trnN-GUU*, *trnP-GGG*, *trnP-UGG*, *trnQ-UUG (x2)*, *trnR-ACG*, *trnR-UCU*, *trnS- GCU*, *trnS-GGA trnS-UGA*, *trnT-GGU*, *trnT- UGU*, *trnV-GAC*, trnV-UAC*, *trnW-CCA*, *trnY-GUA*
	Ribosomal RNA	*rrn16*, *rrn23*, *rrn5*, *rrn4*.*5*
	Small subunit of ribosome	*rps2*, *rps3*, *rps4*, *rps7*, *rps8*, *rps11*, rps12**, *rps14*, *rps15*, *rps18*, *rps19*
	Large subunit of ribosome	*rpl14*, rpl16*, rpl2*, *rpl20*, *rpl22*, *rpl23*, *rpl32*, *rpl33*, *rpl36*
	DNA-dependent RNA polymerase	*rpoA*, *rpoB*, rpoC1*, *rpoC2*
	Translational initiation factor	*infA*
Genes for photosynthesis	Rubisco	*rbcL*
	Photosystem I	*psaA*, *psaB*, *psaC*, *psaI*, *psaJ*, *psaM*
	Photosystem II	*psbA*, *psbB*, *psbC*, *psbD*, *psbE*, *psbF*, *psbH*, *psbI*, *psbJ*, *psbK*, *psbL*, *psbM*, *psbN*, *psbT*, *psbZ*
	ATP synthase	*atpA*, *atpB*, *atpE*, atpF*, *atpH*, *atpI*
	Subunits of cytochrome	*petA*, petB*, petD*, *petG*, *petL*, *petN*
	Chlorophyll biosynthesis	*chlB*, *chlL*, *chlN*
	NADH dehydrogenase	ndhA*, ndhB*, *ndhC*, *ndhD*, *ndhE*, *ndhF*, *ndhG*, *ndhH*, *ndhI*, *ndhJ*, *ndhK*
Other genes	Maturase	*matK*
	Protease	*clpP*
	Envelope membrane protein	*cemA*
	Subunit of acetyl-CoA	*accD*
	C-type cytochrome synthesis gene	*ccsA*
Genes of unknown function	Conserved open reading frames	*ycf1*, *ycf2*, ycf3**, *ycf4*

The sequenced pt genomes of *Juniperus* species encoded 119 genes, including 82 protein-coding genes, 33 transfer RNA (tRNA) genes, and 4 ribosomal RNA (rRNA) genes (Table 2). Among the 119 identified genes, 16 (8 protein-coding genes and 6 tRNA genes) contained one or two introns; two tRNA genes were duplicated. The *trnA-UGC*, *trnG-UCC*, *trnI-GAU*, *trnK-UUU*, *trnL-UAA*, *trnV-UAC*, *rpl16*, *rpl2*, *rpoC1*, *atpF*, *petB*, *petD*, *ndhA*, and *ndhB* genes contained one intron, whereas *rps12* and *ycf3* contained two introns; two tRNA genes (*trnI-CAU* and *trnQ-UUG*) were duplicated (Table 3).

### Comparative analysis of the plastomes of *Juniperus* species

**Fig 2:**
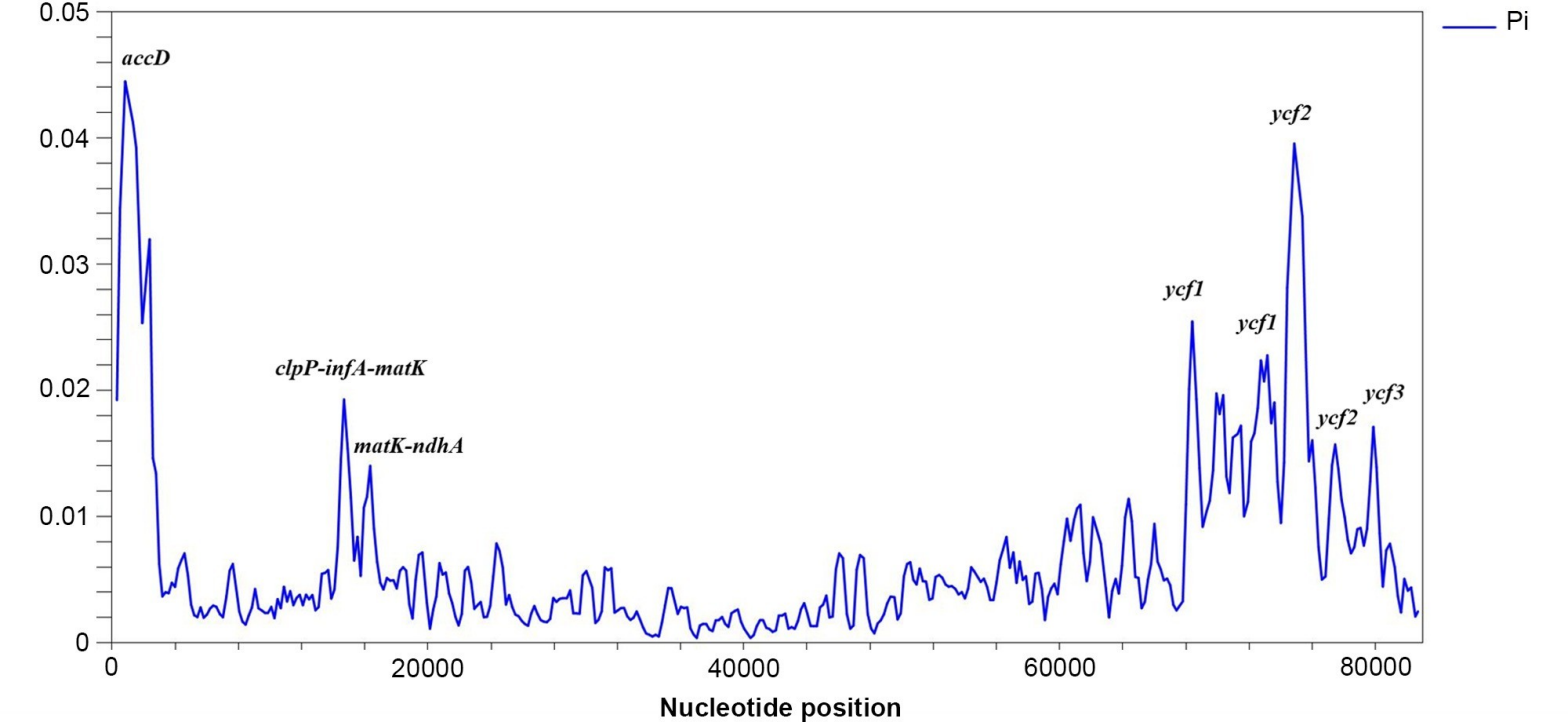
Nucleotide diversity (Pi) in the 82 protein-coding genes of *Juniperus* species. Sliding window analysis with a window length of 600 bp and a step size of 200 bp.

The Pi values for the 82 analyzed protein-coding genes of the seven *Juniperus* complete pt genomes varied from 0.00036 to 0.04373, with an average value of 0.006. The assessment of the variable regions allowed us to identify *accD*, *clpP-infA-matK*, *ycf1*, *ycf2*, and *ycf3* as the five most polymorphic genes (Pi > 0.013). The highest Pi value (0.044) was found in the *accD* gene (Fig 2). In several genes, more than one variable hotspot was identified. For instance, in *accD*, *matK*, and *ycf2*, two variable hotspots were detected, and *ycf1* included more than three variable hotspots (Fig 2).

### Phylogenetic analysis

**Fig 3:**
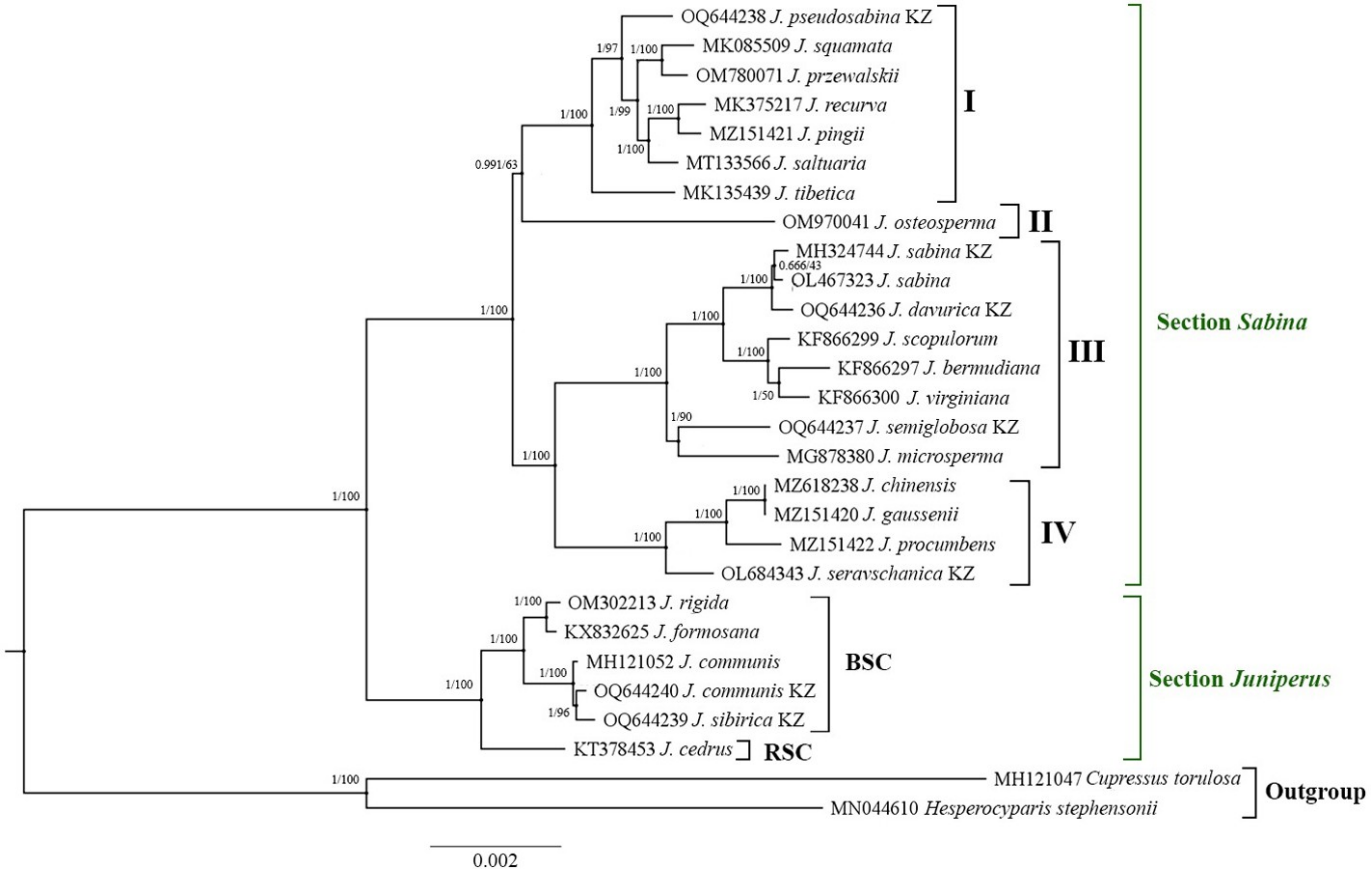
Phylogenetic tree from BI and ML analyses based on 82 shared protein-coding genes of the *Juniperus* and outgroup samples. The numbers above the branches indicate BI/ML support values. Number of clades provided according to Mao et al. (2010) [41].

A phylogenetic tree was constructed based on the concatenated sequences of the 82 common protein-coding genes acquired from the samples of 21 *Juniperus* species from the NCBI database and 5 samples from this study using the BI and ML analyses. *Hesperocyparis stephensonii* was applied as an outgroup in the analysis (Fig 3). The aligned length of the 82 protein-coding genes of the *Juniperus* samples, including the outgroup, was 82,953 bp. In general, 2990 bp (3.6%) of the nucleotide sequences of the coding genes out of the 82,953 bp aligned lengths were polymorphic.

The phylogenetic tree separated the *Juniperus* samples into two major clades, corresponding to the *Juniperus* and *Sabina* sections. Two *Juniperus* species, *J*. *communis* and *J*. *sibirica–*both collected in Kazakhstan were clustered in the clade of the *Juniperus* section. The remaining five species were grouped in different subclades in the clade of the *Sabina* section (Fig 3).

Since the Pi values for the 82 identified genes in seven plastid genomes suggested that the most polymorphic genes were *accD* and *ycf2* (Fig 2), these two genes were applied to reconstruct the phylogenetic tree for samples included in Fig 3. The BI output for the phylogenetic analysis of 26 taxa using *accD* and *ycf2* (S2 Fig) showed a high similarity with the results in Fig 3.

### SSRs and repeat sequences

**Fig 4:**
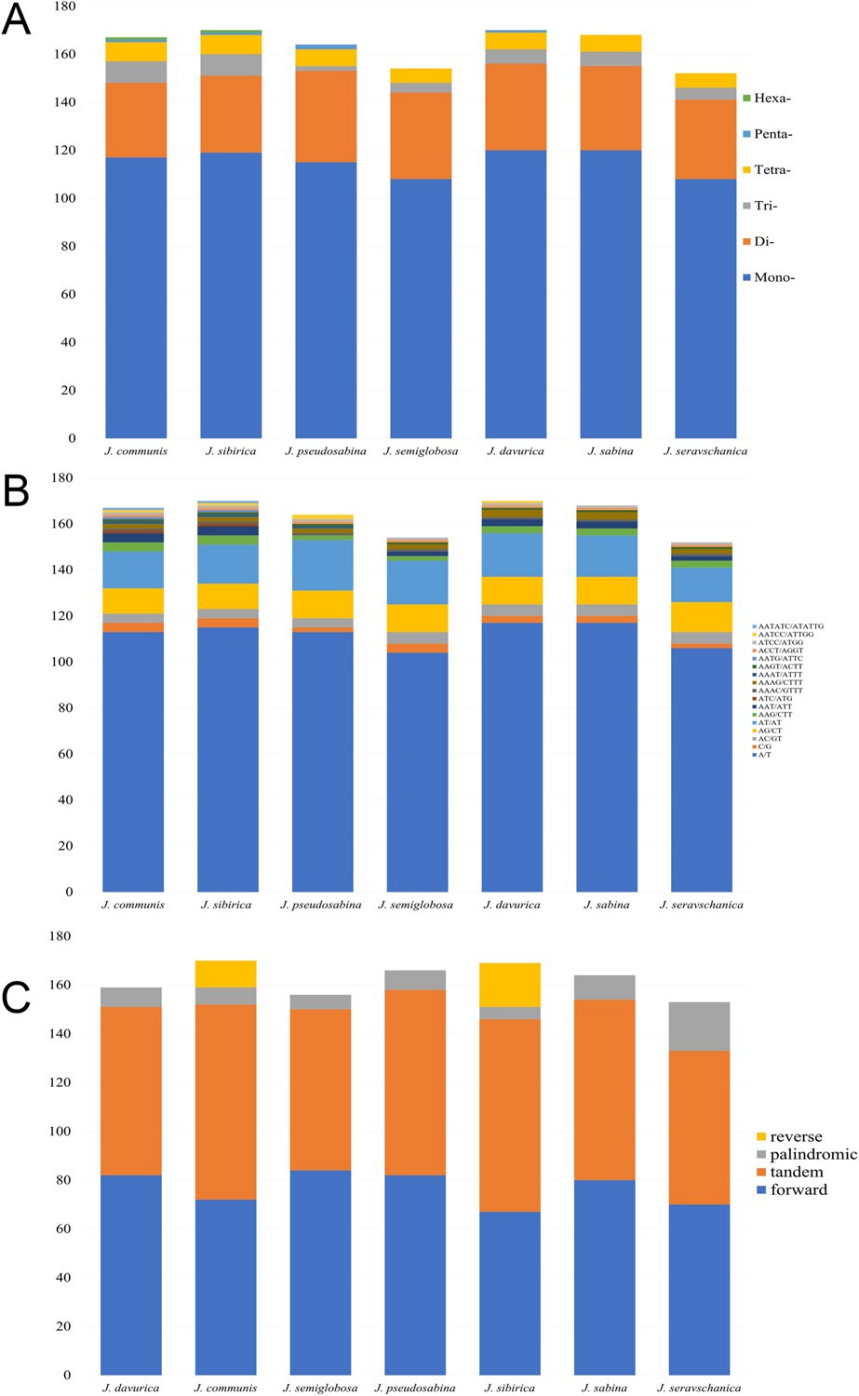
Repeat sequence analysis of seven *Juniperus* plastomes. (A) Number of repeat types; (B) Frequencies of detected SSRs; (C) Number of palindromic, forward, reverse, and tandem repeats.

In total, 1145 SSRs, or microsatellites, were identified in the seven *Juniperus* pt genomes using MISA software. The number of SSRs in individual *Juniperus* pt genomes ranged from 152 in *J*. *seravschanica* to 170 in *J*. *sibirica* and *J*. *davurica* (Table 4). Mononucleotide repeats (807) were the most common motifs in all the analyzed *Juniperus* species, with an average of 70.5% of the total amount of SSRs, followed by 241 dinucleotide (21%), 41 trinucleotide (3.6%), 49 tetranucleotide (4.3%), 5 pentanucleotide (0.4%), and 2 hexanucleotide (0.2%) repeats. The hexanucleotide repeats (AATATC/ATATTG) were least frequent in all seven pt genomes, appearing only in *J*. *communis* (1) and *J*. *sibirica* (1) species (Fig 4A). Most of the mononucleotide repeats were represented by the A/T motif in each plastome; the dinucleotide repeat was represented by the AT/AT motif, the trinucleotide repeat by AAG/CTT, the tetranucleotide repeat by AAAG/CTTT, and the pentanucleotide repeat by AATCC/ATTGG (Fig 4B).

**Table 4:** Types and amounts of simple sequence repeat markers (SSRs) in the pt genomes of *Juniperus* species.

Type	Repeat Unit	Amount							Total
		*J. communis*	*J. sibirica*	*J. davurica*	*J. pseudosabina*	*J. semiglobosa*	*J. sabina*	*J. seravschanica*	
Mono-	A/T	113	115	117	113	104	117	106	785
	C/G	4	4	3	2	4	3	2	22
Di-	AC/GT	4	4	5	4	5	5	5	32
	AG/CT	11	11	12	12	12	12	13	83
	AT/AT	16	17	19	22	19	18	15	126
Tri-	AAG/CTT	4	4	3	2	2	3	3	21
	AAT/ATT	4	4	3		2	3	2	18
	ATC/ATG	1	1						2
Tetra-	AAAC/GTTT	1	1	1	1	1	1	1	7
	AAAG/CTTT	2	2	3	2	2	3	2	16
	AAAT/ATTT	1	1		1				3
	AAGT/ACTT	1	1	1	1	1	1	1	7
	AATG/ATTC	1	1						2
	ACCT/AGGT	1	1	1	1	1	1	1	7
	ATCC/ATGG	1	1	1	1	1	1	1	7
Penta-	AATCC/ATTGG	1	1	1	2				5
Hexa-	AATATC/ATATTG	1	1						2
Total		**167**	**170**	**170**	**164**	**154**	**168**	**152**	**1145**

Repetitive regions, including forward, reverse, palindromic, and tandem repeats in the seven *Juniperus* plastomes were identified using REPuter and Tandem Repeats Finder, respectively. A total of 1137 repetitive regions were detected, including 537 forward, 29 reverse, 64 palindromic, and 507 tandem repeats. Reverse repeats were rare in *Juniperus* plastomes and were found only in *J*. *communis* (11) and *J*. *sibirica* (18). The forward and tandem repeats were widely distributed across the *Juniperus* plastomes, with forward repeats ranging from 64 in *J*. *sibirica* to 84 in *J*. *semiglobosa*, and tandem repeats ranging from 63 in *J*. *seravschanica* to 80 in *J*. *communis* (Fig 4C).

## Discussion

In this study, we have summarized our analysis of the complete pt genome sequences of seven *Juniperus* species from Kazakhstan, including *J*. *sabina* and *J*. *seravschanica*, which we have previously reported [52, 53]. The comparative assessment of the seven pt genomes suggested a high degree of similarity, as all species consisted of 119 genes, including 82 protein-coding genes, 33 tRNA genes, and 4 rRNA genes. The same number of genes has been found in *J*. *chinensis*, *J*. *gaussenii*, *J*. *pingii* and *J*. *procumbens* [54], *J*. *microsperma* [47], *J*. *recurva* [46], and *J*. *tibetica* [45]. All the *Juniperus* plastomes included in this study had lost *rps16*, whereas this gene was present in the *J*. *formosana* (KX832625.1) and *J*. *osteosperma* plastomes in GenBank. Similar to in other studies [44–47], the *rps12* and *ycf3* genes included two intronic regions, *trnI-CAU*, and *trnQ-UUG* were duplicated in the *Juniperus* pt genomes. The sequenced genomes in this study were characterized by a lack of inverted repeats, like in other species from the Cupressaceae family [45–47, 54]. This contrasts with the majority of angiosperm chloroplast genomes that are characterized by two inverted repeats, which divide the genome into large and small single-copy regions [69]. The evaluation of 82 protein-coding genes in seven pt genomes suggested that the total length of these genes was 82,953 bp. An assessment of the nucleotide sequences revealed that 3.6% were polymorphic, confirming the conserved nature of the pt genomes at the genus level [70, 71]. The size of the pt genomes of these seven species was within the expected range (127–128 Kb) of other sequenced pt genomes of *Juniperus* members [46, 47, 49]. The GC content ranged from 34.36% to 34.98% (Table 2); the same value has been observed in other *Juniperus* pt genomes, including those of *J*. *cedrus*, *J*. *tibetica*, *J*. *squamata*, *J*. *chinensis*, *J*. *gaussenii*, *J*. *pingii*, *and J*. *procumbens* [44, 45, 48, 54]. Despite the strong similarity of these seven pt genomes, several highly divergent regions, including *accD*, *clpP-infA*, *matK*, *matK-ndhA*, *ycf1*, *ycf2*, and *ycf3*, were identified. In particular, the specific regions of *accD* and *ycf2* were characterized by high levels of nucleotide diversity (Fig 2), confirming the results reported in other studies of *Juniperus* species [54, 72]. Therefore, the highlighted sequence regions of *accD* and *ycf2* could be used as candidate molecular markers for DNA barcoding and phylogenetic analyses.

In addition to the determination of single-nucleotide polymorphic sequences, the comparative assessment of pt genomes is a powerful approach for the identification of informative simple sequence repeat (SSR) markers [73, 74]. SSRs are widely used as molecular markers in population genetics, species identification, and phylogenetic analyses in plants [73, 75, 76]. In this study, the evaluation of the pt genomes of seven *Juniperus* species from Kazakhstan allowed the identification of 1145 SSRs (Table 4). The dominant SSRs were mononucleotides with A/T as the highest content, confirming the results of previous studies [70, 77, 78]. Most of the pt SSRs were located in the *ycf1* gene and intergenic regions (S1 Table). Our study showed that forward repeats (537) were the most abundant compared to reverse (29) and palindromic (64) repeats. The lengths of all three types of repeats ranged from 31 to 261 bp, similar to those in a previously published report by Androsiuk et al. [79]. As in a previous report on *Juniperus* pt genomes [54], a complement repeat was not observed in any of the seven studied *Juniperus* plastomes.

Nucleotide sequences of pt genomes can also be efficiently used in the molecular systematics of *Juniperus* species. *Juniperus* is known as a taxonomically controversial genus because of its diverse morphological characteristics [32, 33]. In most plants, including *Juniperus*, the pt genome is uniparentally inherited [39]. Despite its slower evolution and lack of recombination, it has variable regions, which can potentially resolve the phylogenetic relationships at the family or the genus level [80]. Unfortunately, previous studies in *Juniperus* used sequences of short DNA regions in nuclear and plastid genomes, which may have potentially affected the precision of the taxonomic relationships [18, 54]. Therefore, we reconstructed a phylogenetic tree using the concatenated sequences of 82 protein-coding genes from 26 samples of different *Juniperus* species, including seven species from Kazakhstan and an outgroup (Fig 3). Similar to published reports using plastid and nuclear DNA regions [41, 81], an assessment of the phylogenetic tree in this work allowed the separation of all samples into two clades that corresponded to the *Juniperus* and *Sabina* sections of the genus and confirmed the monophyletic origin of the genus [1, 80]. The phylogenetic tree, which was based on the concatenated sequences of 82 pt genes, grouped all 26 samples into four out of five clades of the *Sabina* section as reported by Mao et al. (2010) [41]. Compared with the classical work of Mao et al. (2010) [41], this study showed several different patterns when assessing *Juniperus*’ taxonomy. First, in clade I, *J*. *tibetica* was found to be a predecessor of *J*. *pseudosabina* (Fig 3), which was correctly shown in Adams and Schwarzbach (2013) [43]. Second, the assessment of clade III suggested that *J*. *davurica* was an ancestral species to *J*. *sabina* (Fig 3). In contrast, Adams and Schwarzbach (2013) [43] suggested otherwise, and data from Mao et al. (2010) [41] did not resolve the difference between these two species. An evaluation of Fig 3 also revealed that *J*. *semiglobosa* and *J*. *microsperma* were two sister species, unlike in previous reports [41, 43] where *J*. *microsperma* was shown to be a predecessor of *J*. *semiglobosa*. Third, clade IV was populated by *J*. *seravschanica* (Fig 3), which was not studied by Mao et al. (2010) [41]. Our phylogenetic tree also suggested that *J*. *seravschanica* was a predecessor species in this clade (Fig 3). Fig 3 shows that *J*. *chinensis* and *J*. *gaussenii* were identified as two sister species. Only 0.4% of the nucleotides in the 82 studied genes were polymorphic, whereas the phylogenetic tree generated by Mao et al. (2010) [41] suggested that *J*. *gaussenii* was older than *J*. *chinensis*. Also, to confirm the robustness of identified divergent regions in plastid genomes (Fig 2), *accD* and *ycf2* were applied to reconstruct the BI phylogenetic tree of 26 taxa and 2 outgroups using 82 genes (Fig 3). The profile of the phylogenetic tree using these two genes (S2 Fig) was nearly similar to the profile of the dendrogram based on concatenated sequences of 82 genes (Fig 3). The result suggests that *accD* and *ycf2* could be efficiently used as candidate molecular markers for DNA barcoding and phylogenetic analyses. Thus, the sequenced plastomes of *Juniperus* species have provided an immense amount of genetic data that will help to improve resolution in the phylogeny of the genus.

## Conclusion

In this study, we annotated the complete pt genomes of seven species of the *Juniperus* genus grown in Kazakhstan, including two pt genomes (*J*. *sabina* and *J*. *seravschanica*) we previously reported. The size of the pt genomes of these seven species was within the expected range (127–128 Kb) of other reported sequences of the genus. The comparative assessment of the seven pt genomes showed that all consisted of 119 genes, including 82 protein-coding genes, 33 tRNA genes, and 4 rRNA genes. The total length of the 82 protein-coding genes in the seven pt genomes was 82,953 bp, in which only 3.6% were polymorphic, confirming the conserved nature of the pt genomes of the genus. Several highly divergent regions, including *accD*, *clpP-infA*, *matK*, *matK-ndhA*, *ycf1*, *ycf2*, and *ycf3*, were identified. The specific regions of *accD* and *ycf2* were characterized by high levels of nucleotide diversity and would be useful for a DNA barcoding assessment of the genus. Our study predicted the availability of 1145 SSRs in the pt genomes of the genus, majority of which were located in the DNA region *ycf1* and in intergenic regions. A phylogenetic tree using 26 samples of different *Juniperus* species, including seven species from Kazakhstan and an outgroup, was constructed using the concatenated sequences of the 82 genes and the BI and ML approaches. The phylogenetic tree allowed the separation of all samples into two clades that corresponded with the *Juniperus* and *Sabina* sections and confirmed the monophyletic origin of the genus. The phylogenetic analysis also highlighted several differences compared with classical taxonomic studies in J. sect. *Sabina*. Thus, the analysis of the complete sequences of seven pt genomes provided valuable data, which could be used in genetics studies of *Juniperus*.

## Supporting information

S1 Fig Individual circular maps for five newly generated pt genomes of *Juniperus* species. (PDF)

S2 Fig Bayesian inference phylogenetic tree of species in the genus *Juniperus* based on concatenated nucleotide sequences of *accD* and *ycf2* genes. (PDF)

S1 Table SSRs identified in *Juniperus* pt genomes. (XLSX)
