# Correction: Comparative analysis of chloroplast genomes of seven *Juniperus* species from Kazakhstan

**DOI:** 10.1371/journal.pone.0318596

**Published:** 2025-01-28

**Authors:** The PLOS One Staff

There are errors in the Author Contributions. The correct contributions are:

**Conceptualization:** Shyryn Almerekoa, Saule Abugalieva, Yerlan Turuspekov.

**Data curation:** Shyryn Almerekoa

**Formal analysis:** Shyryn Almerekoa, Moldir Yermagambetova

**Funding acquisition**: Shyryn Almerekoa

**Investigation:** Shyryn Almerekoa**,** Moldir Yermagambetova, Smatulla Jumanov.

**Methodology:** Shyryn Almerekoa

**Project administration:** Shyryn Almerekoa**,** Saule Abugalieva.

**Resources:** Shyryn Almerekoa**,** Smatulla Jumanov.

**Supervision:** Shyryn Almerekoa**,** Yerlan Turuspekov.

**Validation:** Shyryn Almerekoa**,** Saule Abugalieva.

**Writing–original draft:** Shyryn Almerekoa**,** Saule Abugalieva, Yerlan Turuspekov.

**Writing–review & editing:** Shyryn Almerekoa**,** Moldir Yermagambetova, Smatulla Jumanov, Saule Abugalieva, Yerlan Turuspekov.

The publisher apologizes for the error.
