# Supplementary material for: Comparative analysis of chloroplast genomes of seven Juniperus species from Kazakhstan
Source: PLoS One. 2024 Jan 25;19(1):e0295550. doi: 10.1371/journal.pone.0295550 (PMC10810545; doi:10.1371/journal.pone.0295550)
Supplement: S1 Fig — (PDF) [file pone.0295550.s001.pdf]

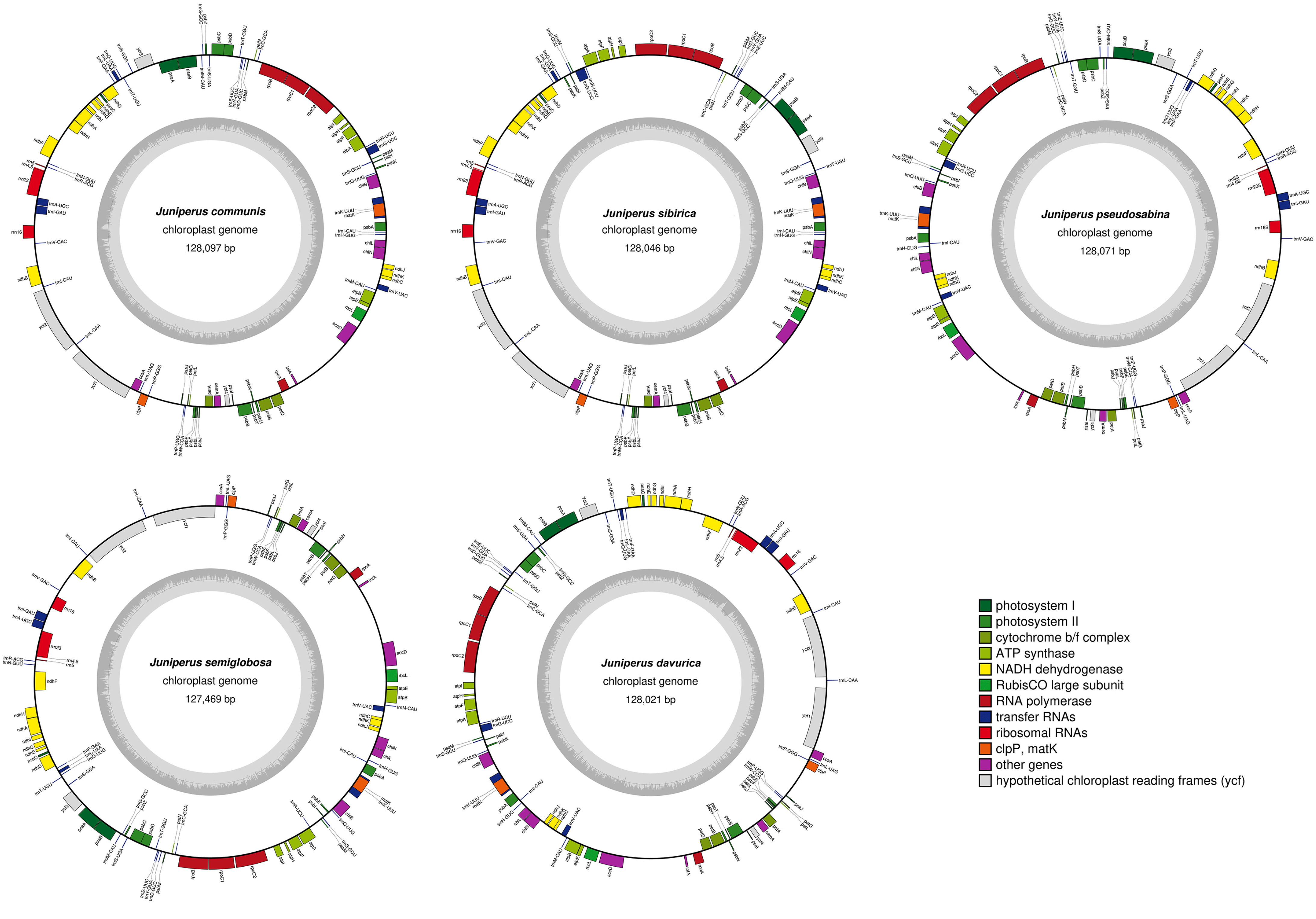
Juniperus communis
chloroplast genome
128,097 bp
Juniperus sibirica
chloroplast genome
128,046 bp
Juniperus pseudosabina
chloroplast genome
128,071 bp
Juniperus semiglobosa
chloroplast genome
127,469 bp
Juniperus davurica
chloroplast genome
128,021 bp
photosystem I
photosystem II
cytochrome b/f complex
ATP synthase
NADH dehydrogenase
RubisCO large subunit
RNA polymerase
transfer RNAs
ribosomal RNAs
clpP, matK
other genes
hypothetical chloroplast reading frames (ycf)
