# Supplementary material for: Comparative analysis of chloroplast genomes of seven Juniperus species from Kazakhstan
Source: PLoS One. 2024 Jan 25;19(1):e0295550. doi: 10.1371/journal.pone.0295550 (PMC10810545; doi:10.1371/journal.pone.0295550)
Supplement: S2 Fig — (PDF) [file pone.0295550.s002.pdf]

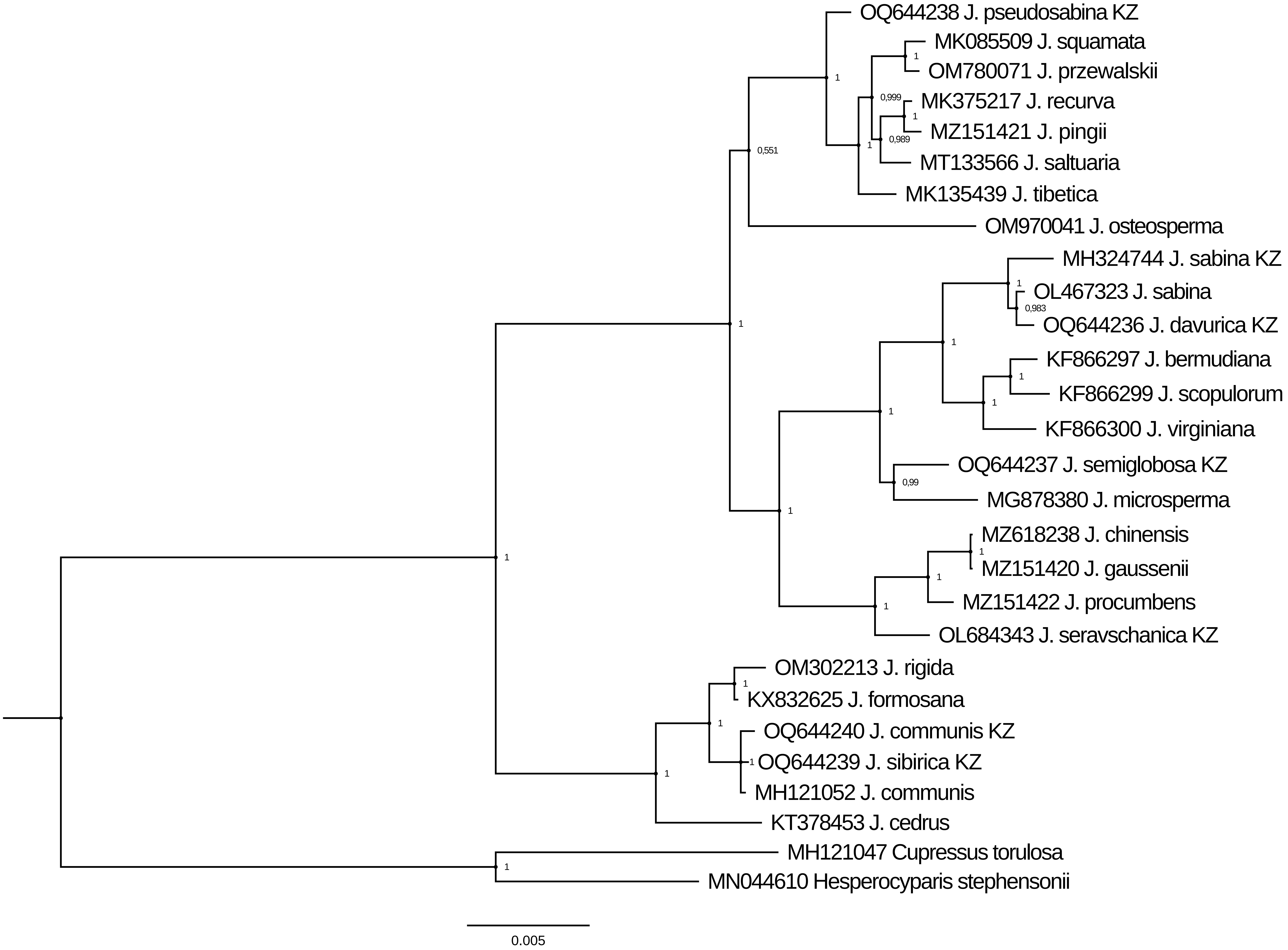
OQ644238 J. pseudosabina KZ
MK085509 J. squamata
OM780071 J. przewalskii
MK375217 J. recurva
MZ151421 J. pingii
MT133566 J. saltuaria
MK135439 J. tibetica
OM970041 J. osteosperma
MH324744 J. sabina KZ
OL467323 J. sabina
OQ644236 J. davurica KZ
KF866297 J. bermudiana
KF866299 J. scopulorum
KF866300 J. virginiana
OQ644237 J. semiglobosa KZ
MG878380 J. microsperma
MZ618238 J. chinensis
MZ151420 J. gaussenii
MZ151422 J. procumbens
OL684343 J. seravschanica KZ
OM302213 J. rigida
KX832625 J. formosana
OQ644240 J. communis KZ
OQ644239 J. sibirica KZ
MH121052 J. communis
KT378453 J. cedrus
MH121047 Cupressus torulosa
MN044610 Hesperocyparis stephensonii
1
0,999
0,989
0,551
0,983
0,99
0.005
